# Editorial: The Role of Epigenetic Modifications in Cancer Progression

**DOI:** 10.3389/fonc.2020.617178

**Published:** 2021-01-08

**Authors:** Atsushi Fujimura, Hailong Pei, Hongquan Zhang, Hanna Lucie Sladitschek, Lei Chang

**Affiliations:** ^1^ Department of Cellular Physiology, Okayama University Graduate School of Medicine, Dentistry, and Pharmaceutical Sciences; Neutron Therapy Research Center, Okayama University, Okayama, Japan; ^2^ State Key Laboratory of Radiation Medicine and Protection, School of Radiation Medicine and Protection, Collaborative Innovation Center of Radiation Medicine of Jiangsu Higher Education Institutions, Medical College of Soochow University, Suzhou, China; ^3^ Department of Human Anatomy, Histology and Embryology, Key Laboratory of Carcinogenesis and Translational Research, Ministry of Education, and State Key Laboratory of Natural and Biomimetic Drugs, Peking University Health Science Center, Beijing, China; ^4^ Department of Molecular Medicine, University of Padua, Padua, Italy

**Keywords:** chromatin remodeling, histone modifications, noncoding RNAs, microRNA, long noncoding RNA

Epigenetics describes multimodal molecular mechanisms that confer a certain phenotype on cell without a change in genotype (1). Chromatin remodelers (e.g. SWI/SNF complex) control opening and shutting a gateway for transcription factors to access genomic loci, and chemical modifications on chromatins (e.g. DNA methylation and histone modifications) regulate transcriptional activities, both resulting in a large-scale alteration of transcriptional landscape (2, 3). Small noncoding RNAs also modulate a transcriptome by targeting a large number of sequence-matched mRNAs and sustain an epigenetic state by triggering and maintaining epigenetic gene silencing of chromatin modifier (4).

In the post-genomic era, epigenetic regulations have been widely investigated to understand the molecular mechanisms underlying transcriptional addiction and phenotypic diversity in cancer cells. In this Research Topic, we have organized a collection of reviews and original research articles that shed light on the epigenetic regulations of cancer cell identity and fate. Chemical modifications in histone proteins, such as acetylation, methylation, phosphorylation, and ubiquitination, alter the structure of chromatin and renew the transcriptional landscape. Since the manner of histone modifications is often dysregulated in cancer, understanding the molecular mechanism by which the modifications contribute to cancer progression is important. Illiano et al. describe that targeting histone lysine demethylases (KDMs) JMJD3/UTX complex by chemical inhibitor, GSKJ4 reduces proliferation activity by downregulating CREB stability in acute myeloid leukemia cells. Lin et al. report that a histone demethylase KDM5c, which specifically demethylates trimethylated and dimethylated H3K4, promotes proliferation activity in colon cancer cells.

During the acquisition of malignant traits, such as metastatic capacity, stemness/dormancy, and therapy-resistance, cells become addicted to a transcriptional mode that is peculiar to cancer. As described by Carvalho and supported by a growing body of evidence, tumor initiation is closely associated with the acquisition of a stem-like state (5). Oncogenic signals drive the rewiring of transcriptional networks and epigenetic states in cells, resulting in a constitutively active expression of stem cell-related genes, such as SOX2, MYC, NANOG, etc. BRACHYURY is one of the well-known stem cell factors. Chen M. et al. introduce the molecular regulation and biological aspects of BRACHYURY in cancer cells, and summarize the clinical relevance in various kinds of cancer. Xu et al. report that BRACHYURY regulates cell cycle and apoptotic cell death program. In the regulation of a dormant state of cancer cells, Guo et al. describe that ARF-like GTPase 14 (ARL14) controls dormancy in lung adenocarcinoma. Although the concept of dormancy is controversial in cancer biology, there is an increasing body of evidence that cancer stem cells go to a dormant state in a certain niche and several cues awake them to reactivate cancer stem cell-related traits (6). This concept explains, for example, the late-onset bone metastasis from breast cancer.

Cancer stem cells have been identified by the specific expressions of marker protein (e.g. CD133 in glioma, CD44 in breast cancer, etc.) and characterized with self-renewal capacity, tumor-initiating potential, and their malignant behaviors (e.g. epithelial-mesenchymal transition (EMT), metastasis, therapy-resistance, etc.). In the process of the acquisition of the cancer stem cell-related traits, epigenetic reprogramming is considered to play pivotal roles. Shen et al. report that ALKBH4 acts as a suppressor of EMT program and reduces invasion and metastasis of cancer cells. Mechanistically, ALKBH4 controls the levels of histone H3K4me3 modification by interacting with a methyltransferase WDR5.

Therapy-resistance is a clinically important aspect of cancer stem cells. Romero-Garcia et al. describe the roles of DNA methylation in chemoresistance and a wide variety of modified genomic loci in several types of cancer, and summarize the anti-cancer drugs related to the resistance triggered by the methylations. Quagliano et al. describe the hallmarks of epigenetic alteration-induced therapy resistance, and introduce several chemical compounds that inhibit the activities of epigenetic modifiers. They further summarize the possible effects of these inhibitors and the molecular targets in various types of cancer. Chen Q. et al. describe that Annexin A6 (ANXA6) contributes to radioresistance in nasopharyngeal cancer. Mechanistically, ANXA6 inhibits PI3K/AKT/mTOR signaling pathway and thus induces cell-protective autophagy. Chen B. et al. describe that gene silencing or chemical inhibition of CDK8 reduces radioresistance in colorectal cancer.

Implications of RNAs in epigenetic regulations are widely recognized in cancer biology. For example, modifications on RNAs are emerging hallmarks of the genes under epigenetic control. N^6^-methyladenosine modification in mRNAs promotes the stability and translation efficiency of these mRNAs, resulting in a dynamic alteration of gene expressions and, thus, cancer cell behavior. The process of this modification and the mode of gene expression are similar to those of DNA modifications: there are Writers, Erasers, and Readers regarding N^6^-methyladenosine. Lu et al. describe the molecular process in detail and summarize the impacts in the development of hepatocellular carcinoma. Yu et al. describe each modifier’s characteristic in detail and summarize their implications in various types of cancer. Noncoding RNAs are also important for cancer epigenetics. Xu et al. describe that long noncoding RNA LINC-PINT suppresses cellular proliferation of melanoma cells. They provide the evidence that LINC-PINT recruits a histone methylase EZH2 (enhancer of zeste homolog 2) to the loci of proliferation-related genes like PCNA. Zou et al. describe the significance of single-nucleotide variants in long intergenic non-protein coding RNAs. Wang et al. demonstrate microRNA miR-27a targets EGFR and its phosphorylation, resulting in suppression of cutaneous squamous cell carcinoma. As one of the emerging evidence that RNA editors are important for epigenetic regulation in cancer, Chen J. et al. report a large number of RNA editing sites, which are associated with hepatocellular carcinoma.

## Author Contributions

AF and LC wrote the manuscript. All authors contributed to the article and approved the submitted version.

## Funding

LC is supported by National Natural Science Foundation of China (Grants No: 31971165), the Fok Ying-Tong Education Foundation, China (Grants No: 171017), the Natural Science Foundation of Jiangsu Province (Grants No: KB20191422). AF is supported by Grant-in-Aid for Scientific Research from the Ministry of Education, Culture, Sports, Sciences, and Technology of Japan (20K07618) and the Japan Agency for Medical Research and Development (AMED) (18072932 and 20318557) (AF).

## Conflict of Interest

The authors declare that the research was conducted in the absence of any commercial or financial relationships that could be construed as a potential conflict of interest.
